# Non-optimal apparent temperature and cardiovascular mortality: the association in Puducherry, India between 2011 and 2020

**DOI:** 10.1186/s12889-023-15128-6

**Published:** 2023-02-08

**Authors:** Shreya S. Shrikhande, Hugo Pedder, Martin Röösli, Mohamed Aqiel Dalvie, Ravivarman Lakshmanasamy, Antonio Gasparrini, Jürg Utzinger, Guéladio Cissé

**Affiliations:** 1 Swiss Tropical and Public Health Institute, Kreuzstrasse 2, CH-4123 Allschwil, Switzerland; 2 University of Basel, Basel, Switzerland; 3 Population Health Sciences, University of Bristol, Bristol, UK; 4 School of Public Health and Family Medicine, Centre for Environmental and Occupational Health Research, University of Cape Town, Cape Town, South Africa; 5 State Surveillance Officer, Department of Health and Family Welfare Services, Govt. of Puducherry, Puducherry, India; 6 Department of Public Health, Environments and Society, London School of Hygiene and Tropical Medicine, London, UK; 7 Centre On Climate Change and Planetary Health, London School of Hygiene and Tropical Medicine, London, UK; 8 Centre for Statistical Methodology, London School of Hygiene and Tropical Medicine, London, UK

**Keywords:** Climate change, Temperature, Cardiovascular disease, LMIC, Adaptation, Modelling, India, Puducherry

## Abstract

**Background:**

Cardiovascular diseases (CVDs), the leading cause of death worldwide, are sensitive to temperature. In light of the reported climate change trends, it is important to understand the burden of CVDs attributable to temperature, both hot and cold. The association between CVDs and temperature is region-specific, with relatively few studies focusing on low-and middle-income countries. This study investigates this association in Puducherry, a district in southern India lying on the Bay of Bengal, for the first time.

**Methods:**

Using in-hospital CVD mortality data and climate data from the Indian Meteorological Department, we analyzed the association between apparent temperature (T_app_) and in-hospital CVD mortalities in Puducherry between 2011 and 2020. We used a case-crossover model with a binomial likelihood distribution combined with a distributed lag non-linear model to capture the delayed and non-linear trends over a 21-day lag period to identify the optimal temperature range for Puducherry. The results are expressed as the fraction of CVD mortalities attributable to heat and cold, defined relative to the optimal temperature. We also performed stratified analyses to explore the associations between T_app_ and age-and-sex, grouped and considered together, and different types of CVDs. Sensitivity analyses were performed, including using a quasi-Poisson time-series approach.

**Results:**

We found that the optimal temperature range for Puducherry is between 30°C and 36°C with respect to CVDs. Both cold and hot non-optimal T_app_ were associated with an increased risk of overall in-hospital CVD mortalities, resulting in a U-shaped association curve. Cumulatively, up to 17% of the CVD deaths could be attributable to non-optimal temperatures, with a slightly higher burden attributable to heat (9.1%) than cold (8.3%). We also found that males were more vulnerable to colder temperature; females above 60 years were more vulnerable to heat while females below 60 years were affected by both heat and cold. Mortality with cerebrovascular accidents was associated more with heat compared to cold, while ischemic heart diseases did not seem to be affected by temperature.

**Conclusion:**

Both heat and cold contribute to the burden of CVDs attributable to non-optimal temperatures in the tropical Puducherry. Our study also identified the age-and-sex and CVD type differences in temperature attributable CVD mortalities. Further studies from India could identify regional associations, inform our understanding of the health implications of climate change in India and enhance the development of regional and contextual climate-health action-plans.

**Supplementary Information:**

The online version contains supplementary material available at 10.1186/s12889-023-15128-6.

## Background

Anthropogenic activity contributes to the accelerating pace of natural climate change leading to an increase in the frequency, intensity and impact of extreme weather events and a global rise in temperatures [1, 2]. The last decade has seen the highest temperatures recorded with 2016 and 2020 emerging as the hottest years on record [3].

Climate change has emerged as a threat to human health and a major public health challenge over the past few decades [4]. Indeed, the recent Global Burden of Disease Study showed that non-optimal temperatures are now among the top-10 leading causes of death globally [5]. Climate change and health research has recently expanded to include direct and indirect effects of temperature, mainly heat, on non-communicable diseases (NCDs) such as cardiovascular diseases (CVDs) [6–8].

CVDs is an umbrella term for a multifactorial group of diseases affecting the structure and function of the heart. They are the leading cause of death globally, claiming an estimated 17.9 million lives each year, accounting for 32% of global deaths [9]. Most CVDs are linked to lifestyle and environmental exposures such as smoking, alcohol and substance abuse, obesity, physical inactivity, stress, unhealthy diets, air pollution and noise. In addition, ethnicity, biological sex and age are also important factors driving the risk for the development of CVDs [9, 10].

CVDs are also climate-sensitive, with the risk of mortality or severe illness exacerbating with very high and low ambient temperatures [7, 11–14]. There are several mechanisms postulated to explain the increased risk of temperature-CVD mortality. The cardioregulatory response to heat stress involves an increase in peripheral circulation to allow for thermoregulation along with an increase in core body temperature. When the cardiac output cannot compensate for this, it results in heat intolerance leading to a CV event [10, 14]. Meanwhile, the cardiac workload increases as a response to cold, along with a sustained increase in systemic blood pressure, leading to CV dysregulation, primarily through vasoconstriction, which reduces both blood flow and oxygen supply to the heart [15].

The effects of temperature on CVDs are global, although the exact relationship varies by region, climate and population [7, 16]. A systematic review found that out of 34 studies on temperature-CVD associations, two-thirds (64%) were conducted in high-income countries with little research from low- and-middle income countries (LMICs), most of it being from The People’s Republic of China [7]. With a lower adaptive capacity and relative lack of resources to face the challenge, understanding the regional temperature-CVD association in LMICs is a priority research area, especially as the burden of both extreme temperatures and CVDs is projected to increase in the future [17, 18].

India has already seen an increase in the burden of CVDs over the past decade with CVDs now being the leading cause of mortality and a major public health problem. Ischemic heart disease (IHD) has seen a 40% rise in the number of deaths reported between 2009 and 2019 [19]. Related CVDs like strokes or co-morbidities like diabetes have also become more prevalent over this period [19, 20]. It is therefore important to consider the role temperature might play as a CVD risk factor. The air surface temperature might rise by as much as 4.4 °C by the end of the twenty-first century, based on the Regional Concentration Pathway (RCP) 8.5 scenario, thereby posing a serious threat to many aspects of life, including human health [21].

The climate and socio-cultural diversity is one of the bigger challenges faced when studying climate-health relationships for India. There are six major Köppen-Geiger climate classification zones in India with temperatures ranging from > 0 °C to < 40 °C [22]. The socio-cultural differences between regions and communities is also an important factor determining health and vulnerability. The dense population concentration and movement in urban areas, along with factors such as the urban heat island effect, lead to urban communities, particularly those on the lower socio-economic level in informal settlements, being more vulnerable to climate disasters and heat, despite the relative paucity of cooling facilities in rural areas. [23–25]. Additionally, coastal regions, such as Puducherry, are particularly vulnerable to climate hazards [25, 26]. Given these variations, there is a need to comprehensively study regional CVD-temperature associations in India in order to reduce the burden of CVDs and be prepared for challenges of the future.

We conducted an exploratory study analysing the effects of apparent temperature (T_app_) on CVD mortality in Puducherry, India. To our knowledge, there were no previous region specific studies done on the association between apparent temperature and CVD mortality from this region.

## Methods

Our exploratory study analysed the fraction of in-hospital CVD-related mortalities attributable to T_app_, the so-called ‘fatal admissions’ using a case-crossover design with a distributed lag non-linear model (dlnm).

### Study area

**Fig. 1:**
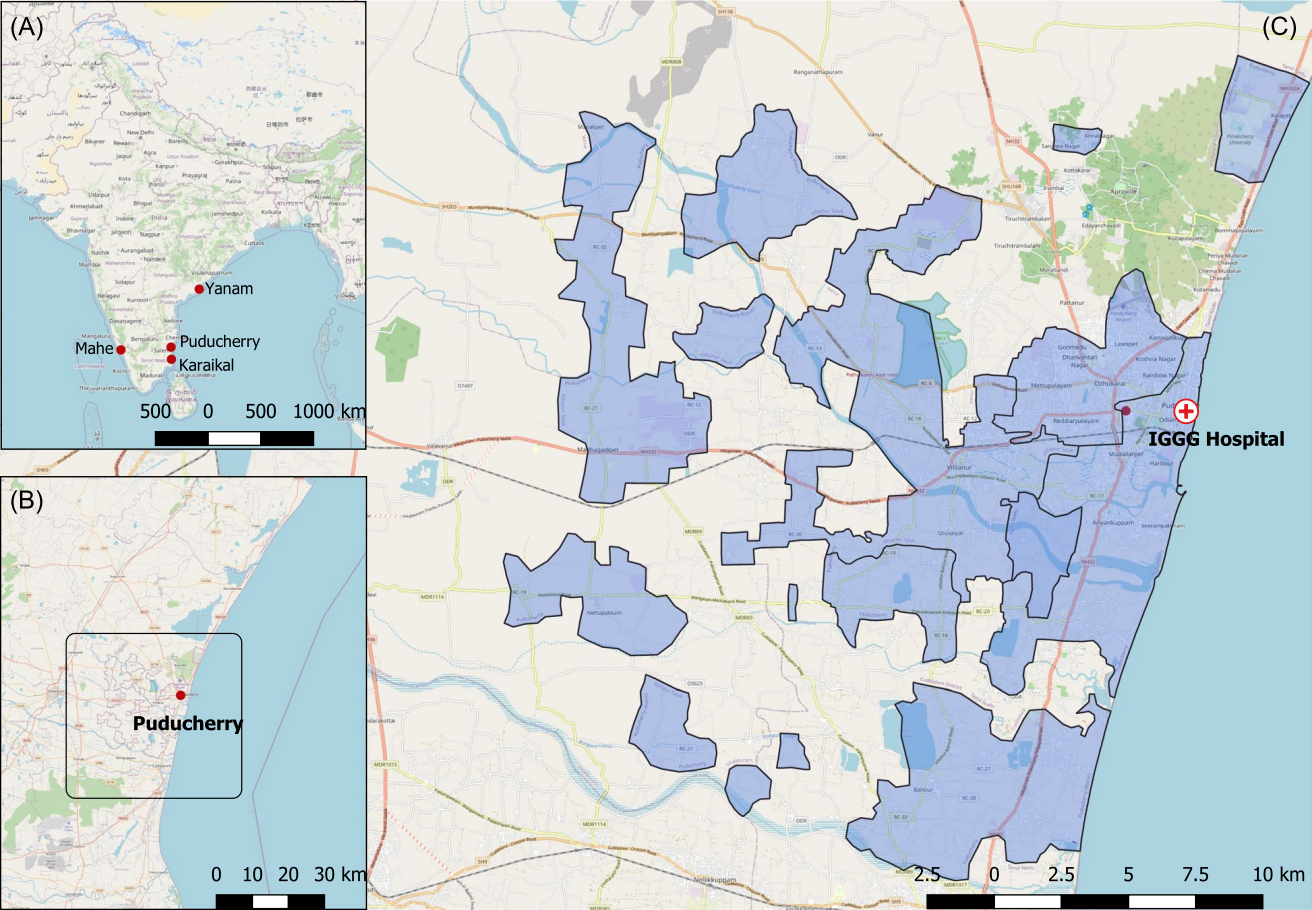
Map showing the four districts of the Union Territory of Puducherry. **A** shows the location of the four districts that make up the Union Territory of Puducherry, namely Puducherry, Karaikal, Mahe and Yanam, spread out on either side of the coast of India. **B** Focuses on Puducherry district which is nestled within the state of Tamil Nadu with Andra Pradesh to the north (inlaid map). The shaded area in panel **C** highlights the non-continuous geographical area of Puducherry district

Puducherry is a unique Union Territory (UT) in India, comprising of four erstwhile French colonies (i.e. Puducherry District, Karaikal, Mahe and Yanam region). Puducherry and Karaikal lie on the eastern coast, within the state of Tamil Nadu, while Yanam, also on the east coast, is surrounded by the state of Andra Pradesh. Mahe lies on the western coast within the state of Kerala. The total area of the UT is 492 km^2^ as seen in Fig. 1A.

This study focuses on Puducherry, which itself covers an area of 294 km^2^ spread out over 4 non-continuous sub-districts or ‘Taluks’ as shown in Fig. 1B and C. As per the Government of India census of 2011, the population of Puducherry is 950,289 with 69.2% of the population residing in urban areas and an almost equal distribution of males and females [27, 28].

Puducherry falls within the tropical savannah with a dry winter climate type as per the Köppen-Geiger classification. The region has a tropical climate with a generally high relative humidity, which is around 80% during October to April and around 70% in June and July. The mean annual temperature is around 30° C.

There are two state government run tertiary care hospitals in Puducherry, out of which only the Indira Gandhi Government General Hospital and Post Graduate Institute (IGGGH) is a large, multispecialty general hospital with a cardiology department. In addition, there is another large, multi-speciality tertiary care hospital, the Jawaharlal Institute of Postgraduate Medical Education and Research. However, this is administered on the central governmental or federal level and due to inaccessibility of health data, our project was limited to IGGGH, serving the entire Puducherry district. It is estimated that between 85–90% of Puducherry’s population is served by IGGGH, as the district headquarters with speciality services available. The remaining 10–15% of the population is likely to seek treatment from JIPMER or other smaller clinics or private health facilities [29].

The unique characteristics of Puducherry added to our interest in focusing on it. First, there are no studies on this topic from this region. Second, as a small area with one main state-government multi-speciality hospital; the quality of the individual level patient data we were allowed access to was suitable for the exploratory study; and finally, as a coastal and one of the most urbanized cities of India, it is more vulnerable to the effects of climate change [25, 28].

### Health data

Daily hospital mortality records were obtained from the IGGGH for the ten year period 2011 to 2020 (*n* = 7,190). The extracted data was de-identified and included information on age, sex, date of admission, date of death, hours spent in hospital before death and cause of death.

For the period 2011–2015, mortality records were only available from the cardiology department (*n* = 633) in a non-digital format with several missing months due to fire damaged files, while records for 2016–2020 were in a digital format and included data from all hospital departments (*n* = 6,552). For this latter period, cases from all other departments that had a CVD involvement were identified and included in the analysis (*n* = 3,327). As cases were not classified by ICD codes, CVD cases were identified with a cardiologist consultant and classified into categories. We made three broad categories based on ICD-10 codes; (i) IHD; (ii) cerebrovascular accidents (CVA); and (iii) other. Other is comprised of cardiopulmonary diseases, hypertensive disorders, peripheral vascular disease, rheumatic heart disease, congenital heart disease, aortopathies and all other CVDs. A total of 3,960 mortality cases over the ten year period with CVD involvement were included in the study. The codebook is presented in the supplementary material Table 1.

Our main analysis was based on the aforementioned individual CVD mortality data. We also obtained the monthly hospital records for the entire hospital and cardiology department showing the total monthly admissions and deaths and presented it graphically to highlight the overall trends in hospital mortalities and admissions (Figure S5). Missing records were assumed to be missing-at-random, since missingness was likely only related to time. As time was accounted for in our model a complete case analysis, one in which the analysis is restricted to all individuals for which data are available, should give unbiased estimates.

### Meteorological data

Daily weather records from two weather stations serving Puducherry (i.e., Puducherry city and Cuddalore) covering the period 2010 to2020, were obtained from the Indian Meteorological Department (IMD). The variables included maximum temperature (T_max_), minimum temperature (T_min_), average wind speed (WS), dry bulb temperature (Ta) and relative humidity (RH).

We chose to use T_app_ as the main exposure variable of interest as it also accounts for the effect of RH and vapour pressure (hPa) along with temperature, thereby better capturing the physiologically ‘felt’ exposure. An average T_app_ for Puducherry was calculated by combining individual station data with the Steadman’s equation [30],as follows:

$${\mathrm{T}}_{\mathrm{APP}}=\mathrm{ Ta}+0.33 \times \mathrm{ hPa}-0.7 \times \mathrm{ WS}-4$$

$$\mathrm{hPa}=\frac{\mathrm{RH}}{100}\times 6.105 \times {\mathrm{e}}^{(\frac{17.27 \times {\mathrm{Ta}}}{237.7 + {\mathrm{Ta}}})}$$

where Ta is the dry bulb temperature (^o^C), hPa is the vapour pressure, RH is the relative humidity (%) and WS is the wind speed (m/s). The daily T_app_ calculated for both weather stations was then grouped by date, from which we calculated a daily average T_app_ for the entire region of Puducherry. This average daily T_app_ was the one included in our model. Comparative figures of the data from individual weather stations is shown in Figure S11 and S12.

### Statistical model

Our model consisted of the self-matched case-crossover design using dlnm, as described in [31, 32] to capture the non-linearity and delayed association between T_app_ exposure and risk of in-hospital CVD mortality (hereon referred to only as CVD mortality). By design, this allows for the estimation of the average ‘within-case’ risk while controlling for between subject time-varying factors [33]. As our dataset consisted only of patients who died in hospital, we chose to use the day of admission as our main ‘event’ since there was no way to determine environmental exposure (presence or absence of air conditioning), medical treatments administered prior to death or other prognostic factors that might have differed between out-of-hospital and in-hospital days. Our study therefore focussed on the association between T_app_ and the risk of ‘fatal CVD admission’ following hospitalisation.

We used a time-stratified approach in which each case of mortality served as its own control, with the comparable control days being matched by the same day of the week within the same month to generate a total sample size of 17,352 (3,960 cases and 13,392 controls) used in the final model. We chose to model the T_app_-CVD mortality risk over 21 days to capture long term lags as well as any short term harvesting effect. We constructed the crossbasis by creating a lag matrix over 21 days using the whole T_app_ series, which was matched with the cases and bi-directionally sampled controls. We modelled the exposure–response association using binomial likelihood distribution with a natural cubic spline with 2 internal knots placed at the 25^th^ and 75^th^ percentile of the T_app_ range. For modelling the lagged-response, we used a natural cubic spline with 3 internal knots placed equally on the log scale to allow for consistency and comparability with similar studies done previously [22]. A time-stratified design was adopted to regulate potential time-invariant confounders (e.g., age and sex) using self-control and limit bias from temporal confounders (e.g. secular trends, seasonality, day of the week effects, etc.) and exclude long-term impact of air pollutants.

We expressed risks in relation to the minimum mortality temperature (MMT). The MMT was derived from the point on the cumulative exposure–response curve with the lowest associated risk of mortality. This value was used to centre the overall cumulative exposure–response and also considered to be the optimal temperature. We have reported our findings as the fraction of CVD mortalities that can be attributed to temperature, or the attributable fraction (AF). The total number of deaths attributable to non-optimal temperatures, both hot and cold was calculated using the MMT as a reference with a backward perspective. The AF is derived as a ratio of the number of deaths attributable to non-optimal temperatures and the total number of deaths [34]. We feel this measure is better suited to show the general trend for how temperature affects CVD mortalities in Puducherry. Empirical confidence intervals have been calculated using Monte Carlo simulations with 1000 replicates. Additionally, to limit spurious values at the extremes we have restricted plots and analyses to the central 95^th^ percentile of the T_app_ distribution.

### Subgroup analysis

#### Age and sex

We performed subgroup analyses with stratifications for age and sex combined. The age categories used were above and below 60 years of age to account for post-menopausal women. 3 cases were missing information on age and were thus excluded from the stratified analysis. A total of 17,338 cases and controls were included in the age-and-sex stratified analysis, out of which 6030 and 11,308 were below and above the age of 60 years respectively.

#### CVD class specific

We stratified the analyses by type of CVD using 3 main classes: (i) IHD; (ii) CVA; and (iii) all other CVDs. Many cases presented with multiple classes of CVDs and since the dataset did not contain ICD-10 codes, there was no way to know the primary cause of death. Such cases were considered in all the CVD classes they presented with and therefore, this cause-specification is patients who died with the particular CVD as opposed to from.

### Sensitivity analysis

Sensitivity analyses were performed to explore the impact of different numbers and placement of knots, changing the regression to quasi-Poisson, excluding patients who stayed in hospital for longer than 10 days and also using only 5 years of data (2016–2020). The results are presented in Supplement figures S1 to S4 and S7.

All data were analysed using a combination of Microsoft Excel 2016 and the R software (version 4.0.3, The R Foundation for Statistical Computing Platform 2020). The main packages used were ‘dlnm’ to fit the dlnm model and ‘attrdl’ for the attributable fraction [31]. The methodology used in this project abided by the principles laid out in the Declaration of Helsinki.

## Results

### Descriptive statistics

As seen in Table 1, out of the 3,960 cases of in-hospital mortality with a CVD involvement between 2011 and 2020 that were included in this study, the average patient spent 4 days in hospital. There is no data on whether these were emergency visits or planned visits. More than half (54%) of patients died within 48 h of being admitted to the hospital.

The mean dry bulb temperature and humidity for Puducherry was around 29 °C and 76%, respectively. The mean T_app_, which takes both of these into account along with VP and WS was slightly higher in Puducherry between 2010 and 2020, around 33 °C. Two thirds of the patients were older than 60 years (65.2%). More males than female patients died of a CVD related mortality. Over 50% of patients in this study also had at least one co-morbidity associated with CVDs, namely hypertension, diabetes or alcoholism.

**Table 1:** Table of descriptive statistics showing the distribution and characterization of the meteorological data and patient data. N/A denotes missing information. Additional information on climate variable distribution by patient characteristics is presented in Table S2

		**Apparent Temperature (°C)**	**Average Temperature (°C)**	**Humidity (%)**
**Climate variables**	**Mean ± SD**	33.5 ± 8.6	28.6 ± 2.5	76.6 ± 3.4
	**Min, Max**	22.9, 41.9	19.6, 36	43, 100
**Patient characteristics**			***n* (% of total)**	**(Mean ± SD, Min, Max)**
	**Gender**	**Male**	2366 (59.7)	
		**Female**	1591 (40.2)	
		**Missing**	3 (0.1)	
	**Age (Years)**	** < 20**	24 (0.6)	65 ± 14.2, 1, 104 (Years)
		**21–40**	236 (5.9)	
		**41–60**	1371 (34.6)	
		**61–80**	1937 (48.9)	
		** > 81**	389 (9.8)	
		**Missing**	3 (0.07)	
	**State**	**Puducherry**	3064 (77.4)	
		**Tamil Nadu**	879 (22.2)	
		**Andra Pradesh**	7 (0.2)	
		**Other/Missing**	10 (0.3)	
	**Time spent in hospital**	** < 48 h**	2121 (53.6)	4 ± 4.95, 1, 82 (Days)
		** > 48 h**	1839 (46.4)	
	**Comorbidities**	**Yes**	2176 (54.9)	
		**No**	1784 (45)	

### Cumulative exposure–response association and attributable fraction

**Fig. 2:**
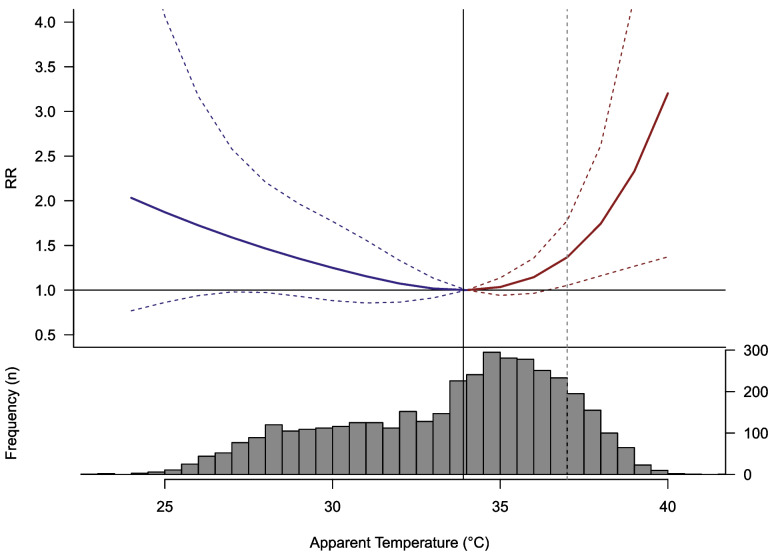
Cumulative apparent temperature (T_app_)-CVD mortality RR with a 21-day lag (dotted lines show the 95% CI) with a histogram of the T_app_ distribution for Puducherry between 2011 and 2020. The black solid vertical line represents the minimum mortality temperature (MMT), while the dotted grey line represents the heat wave threshold at 37 °C. The blue line and red line represent the exposure–response curve for cold and hot temperature relative to the MMT respectively

Figure 2 shows the relative risk (RR) estimates for the association between T_app_ and CVD mortality, cumulatively across the 21-day lag period, with the corresponding T_app_ distribution, MMT and heatwave threshold as defined by the Indian Meteorological Department (IMD) [35].

The cumulative association shows a distinct U-shaped curve with temperatures below and above the MMT showing an increased RR of in-hospital CVD mortality. The MMT itself is 33.9 °C and occurs at around the 60^th^ percentile of the T_app_. The temperature distribution shows that the MMT is close to both the median and mean T_app_ (34.25 °C and 33.5 °C, respectively).

In the 10 year period, the T_app_ was between the MMT and heatwave threshold temperature for 1506 days, representing a total of 41.3% of all days.

The optimal temperature corresponds to the MMT and can be thought of as the temperature with the least associated risk of in-hospital CVD related mortality. Here, all temperatures below and above 34 °C will be considered ‘cold’ and ‘hot’, respectively. While the RR increased rapidly and non-linearly above heatwave threshold temperatures, there are fewer days (496 days) with extremely hot temperatures above the heatwave threshold.

Overall, 17.4% (95% CI 6.4–26%) of the in-hospital CVD related deaths can be attributed to non-optimal temperatures within the study period (Table 2). Out of these, colder non-optimal temperatures, consisting of 1645 days, have a higher burden with 8.3% of deaths (95% CI -2.5–16.6%) attributable to cold as compared to 9.1% (95% CI intervals 0.9–15.8%) of deaths being attributable to heat, representing 2002 days in the study period.

**Table 2:** The overall attributable fraction (AF) for overall non-optimal apparent temperature (Tapp), cold Tapp and hot Tapp in Puducherry with the 95% CI

	**Attributable Fraction (%)** **(95% CI)**
Non-optimal T_app_	17.4 (6.4, 26)
Cold Tapp	8.3 (-2.5, 16.6)
Hot Tapp	9.1 (0.9, 15.8)

### Lagged association

**Fig. 3:**
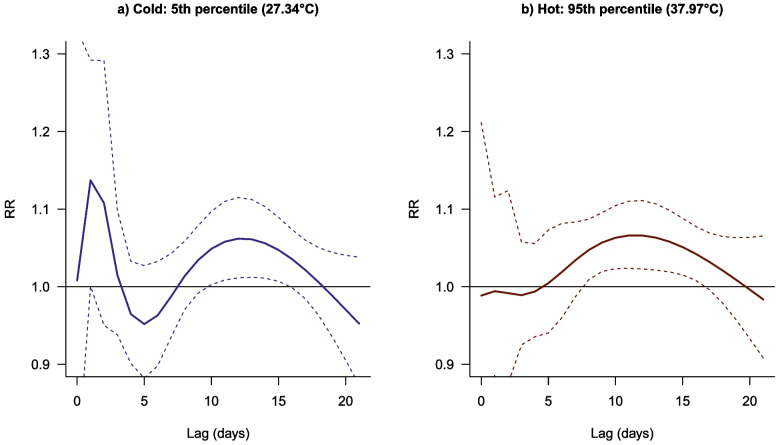
The RR for the lagged apparent temperature (T_app_)-CVD mortality association at the 5^th^ and 95^th^ percentiles of the T_app_ range. (a) The blue line represents the cold temperature at 27.3 °C and (b) The red line represents the hot temperature at 38.0 °C. The dotted lines represent the 95% CI

Figure 3 represents the lagged response association for the 5^th^ (27.3 °C) and 95^th^ (38.0 °C) percentile of the T_app_ distribution over a 21-day period.

Colder temperature has an almost immediate response or increase in RR, while hot temperatures show a delayed association. The cold effect peaks at day 1 before gradually decreasing to a protective risk at lag day 5 (with no statistical significance). The cold-CVD mortality association risk then increases slightly again from around day 7 to day 16 where it peaks around day 11, as seen in Fig. 3a. Hot temperature related risk of CVD-mortality is only seen after a 5-day lag period, with an initial protective effect, and persists for 16 days, although the confidence intervals are quite wide as shown in Fig. 3b. This risk is relatively lesser compared to the cold-CVD mortality risk.

### Age-and-sex stratification

**Fig. 4:**
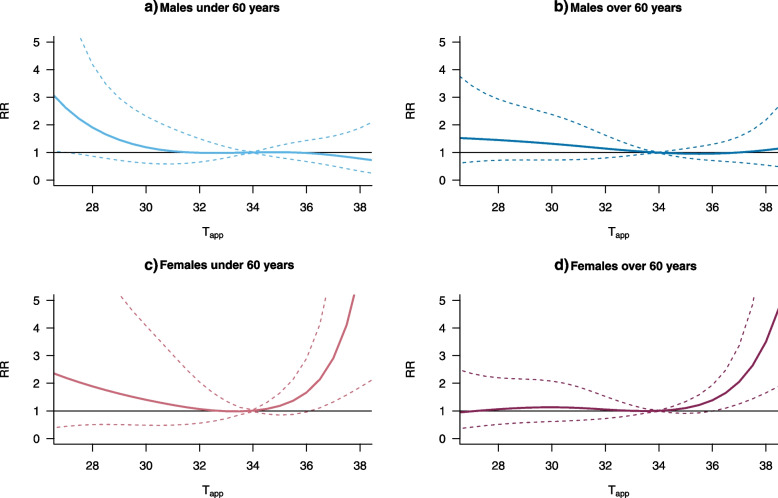
The RR of apparent temperature (T_app_) attributable CVD mortality among **a** males under 60 years, **b** males over 60 years, **c** females under 60 years and **d** females over 60 years. Graphs are restricted to the central 95% of temperature due to wide CIs at the extreme ends

In order to better understand this association in different groups, we performed age-and-sex stratified analyses. Sex and age group both seem to be a contributing factor to the risk temperature related CVD mortality, as seen in Fig. 4.

Males both above and below 60 years of age seem to be largely unaffected by heat (Fig. 4a and b). Females below 60 years of age are affected by both heat and cold, although the heat effect is predominant (Fig. 4c). Females over 60 years are more likely to beunaffected by cold and have a higher risk from heat on average (Fig. 4d).

We found that males of all ages are at a relatively similar risk for temperature attributable CVD mortality (Table 3). Females below 60 years have a higher AF to non-optimal temperatures compared to older females, primarily since they are also sensitive to cold. Females over 60 years are unaffected by cold and their AFs are mainly heat related.

**Table 3:** The fraction of CVD mortality attributable to overall non-optimal apparent temperature (T_app_) cold and hot non-optimal T_app_ for males and females above and below the age of 60 years with the number of cases and controls for each category

Sex and age (years)	AF% (95% CI)	Cold AF% (95% CI)	Hot AF% (95% CI)
Males below 60 (*n* = 4139)	11.81 (-14.88, 27.2) 4.4 (-19.3, 19.5)	15.32 (-7.31, 28.6) 6.8 (-15.9, 20.7)	-3.54 (-23.15, 10.02) -2.4 (-21.8, 12)
Males over 60 (*n* = 6222)	12.85 (-8.62, 26.93) 7.3 (-10.6, 20.3)	16.91 (-3.39, 28.6) 8.3 (-10.1, 21.4)	-4.16 (-19.68, 6.82) -1 (-15.1, 9.8)
Females below 60 (*n* = 1891)	23.21(-32.86, 41.93) 28.4 (-4, 43.3)	3.15 (-56.42, 27.52) 8.8 (-28, 27.2)	20.01 (3.47, 29.38) 19.8 (1.5, 31)
Females over 60 (*n* = 5086)	8.63 (-22.44, 27.47) 16.3 (-4, 28.1)	-6.62 (-46.69, 14.22%) 2.5 (-22, 17.4)	15.2 (5.56, 22.29) 13.8 (3.7, 22.1)

### CVD type stratification

**Fig. 5:**
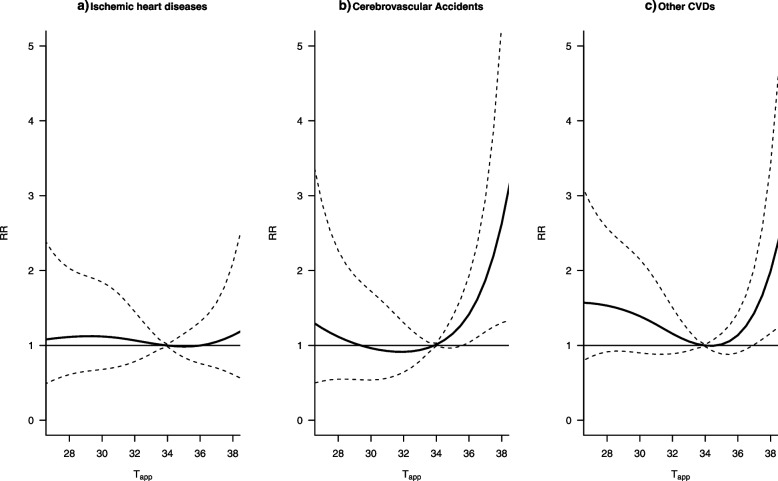
Cause-specific apparent temperature (T_app_)-mortality association for patients who died with (**a**) ischemic heart diseases, (**b**) cerebrovascular accidents and (c) all other types of CVDs. The graphs have been restricted to the central 95th percentile of the T_app_ range

Stratified analyses for the type of CVD revealed that IHDs do not seem to be particularly affected by heat and minimally affected by cold, as shown in Fig. 5a. For CVAs, cold temperatures affect the risk of mortality less than heat, as can be seen in Fig. 5b. All other forms of CVDs seem to display the same U-shaped association seen in the cumulative association and are affected by both heat and cold (Fig. 5c). As such, the results shown here are of patients who died with that particular CVD as opposed to from that particular CVD.

### Sensitivity analysis

The findings from the sensitivity analyses are presented in the Supplementary material (S1 to S4 and S7). Briefly, the associations identified in our main analysis were insensitive to changes explored, though there was some sensitivity of predicted associations at more extreme temperatures, where data available for estimation was more limited.

## Discussion

Our findings show that despite having generally high T_app_ throughout the year, both cold and hot non-optimal temperatures are responsible for contributing to excess CVD deaths in Puducherry. The overall AF we found was 17.4%, with an almost equal burden attributable to cold (8.32%) and hot (9.1%) non-optimal temperature withA review spanning 750 locations across 43 countries found that out of 9.4% of all-cause excess deaths attributable to non-optimal temperatures, 8.5% were cold related while 0.9% were heat related, thereby supporting our findings, especially related to cold [36]. In inherently hot regions like Puducherry, it thus becomes important to consider cold exposure as an important contributor to temperature-related mortality.

We found that cold exposure had a bi-level lagged response with a sharp, immediate increase in mortality risk followed by a protective effect over a short time period before a second peak of increased risk in CVD mortality, most likely due to the long-term effects of cold. On the other hand, heat exposure showed a delayed CVD-response. This differs from other studies, which found an immediate effect due to heat and a more lagged cold response [22, 33, 37, 38]. The harvesting effect or mortality displacement, when the most vulnerable people are affected earlier than the healthier members of the population, thereby bringing mortality forward in time, could explain the immediate increase in cold related mortalities followed by the slightly reduced risk till about lag day 7 [39, 40]. Since the average T_app_ in Puducherry is around 34 °C, the population is likely more adapted to temperatures above 30 °C. Repeated exposures to temperatures above 30 °C could induce a form of thermal pre-conditioning. This sub-lethal, frequent heat exposure could help to build tolerance and confer protection against further lethal thermal stress brought on by extremely high temperatures [13]. The thermal pre-conditioning effect has been found to set in within hours of exposure and can last up to 5 days, potentially explaining the 5-day lag seen for hot temperatures in Puducherry [41].

Additionally, there is a greater proportion of ‘cold’ days compared to ‘hot’ days or ‘extremely hot’ days. For the coastal regions, a heatwave is declared when the maximum temperature rises above 37 °C and is a departure of 4.5 °C or more from the normal temperature, as per the IMD [35]. There are relatively few consecutive ‘extremely hot’ days in Puducherry, while there is often a ‘cold spell’ lasting for several days, especially during the winter months, which could affect the population negatively, especially if they are unaccustomed to it. Indoor heating systems are also uncommon in the southern part of India where temperatures rarely drop below 20 °C. However, the IMD definition of a cold wave in coastal areas is when the minimum temperature is < 15 °C or a departure of 4–5 °C from minimum temperature, meaning that there has been no official cold wave recorded in Puducherry for several years [42, 43]. Puducherry is one of the most urban territories in India with 68.3% of the population considered as urban according to the 2011 Census [28]. Typical urban characteristics that modify the temperature effect on health, such as tightly packed spaces and living quarters, population density, air pollution and green spaces, might contribute to the overall relatively high heat AF we found [44]. Thus, our results also highlight the importance of regionally defining cold and heat from a health perspective using the MMT. The MMT percentile also seems to vary by region, with tropical and subtropical regions having a MMT around the 60th percentile of the temperature distribution compared to the 80th or 90th percentile as seen in temperate regions [45]. Therefore, a one-measure-fits-all approach cannot be used to describe the temperature-CVD mortality or all-cause mortality relationship [46].

We were able to identify the differences in the temperature-CVD mortality association between sexes and age simultaneously, which to our knowledge has not been studied yet in the Indian context. Our results demonstrate that age and sex together act as effect modifiers. All males were more likely to be susceptible to cold compared to heat. Males aged above 60 years were more vulnerable to cold non-optimal temperatures than females in the same age bracket, who were more susceptible to hot non-optimal temperatures and seem to withstand cold better, as a whole. Meanwhile, females below 60 years were affected by both hot and cold non-optimal temperatures. We postulated several possible explanations for this phenomenon. Overall, age is a common risk factor for CVD mortality with older people, especially women, being more susceptible [13, 47–50]. Most women over the age of 50 years have undergone menopausal transition, which has long been associated with decreased cardio-protection and an increase in the risk of developing CVDs and vulnerability to heat [51]. Sex differences in thermoregulation could also be a factor for these findings. For example, the temperature threshold, above which sweating is induced, is higher in women than in men while their overall sweat output is lesser, resulting in reduced heat tolerance [52, 53]. On the other hand, men have found to have a greater decrease in core body temperatures when exposed to cold compared to women, leading to a higher cold intolerance or sensitivity [54, 55]. A study by Achebak et al*.,* in Spain reported a similar relationship between older females and males being more susceptible to CVD mortality from heat and cold respectively [56].The context of Puducherry might also play a role in this association. Manual outdoor labour including agriculture and construction are common occupations for many men, potentially helping them build tolerance to higher temperature. Traditionally, females, especially older females, are more likely to spend a larger part of the day indoors where the urban island effect, inadequate air conditioning and physiological factors could make them more vulnerable to heat [57].

The findings from our cause-specific analysis compare to a recent study by Schulte et al*.,* in Switzerland which found limited risks of mortality from myocardial infarction (part of IHD in our study) associated with temperature [49]. They also found the risk of mortality from strokes (CVA in our study) increases with heat. The findings are also similar to the Fu et al., study from India, which found smaller cold-attributable risk in addition to a U-shaped curve for CVAs as we confirmed [22].

Many studies look at the temperature-mortality association, but few look at CVDs in particular. The MMTs for all-cause mortality are derived as a function of disease-specific MMT [16]. In fact, temperature-CVD mortality associations have been found to be U or J-shaped while various patterns including the inverse U or reverse-J shape have been associated with infectious diseases [33, 58–60]. The association between temperature-CVDs also varies by region and latitude, with different regions within a country reporting different relationships [16, 33, 46, 61]. While most studies have found an increase in CVD events due to heat exposure, a study done across China found that the bigger burden of CVD mortality can be attributed to cold temperatures [33].

As of 2016, 28.1% of all deaths in India were due to CVDs as compared to 15.2% in 1990 and this burden is projected to increase along with the level of epidemiological transition (ETL) [62]. Puducherry falls in the higher-middle ETL bracket with 53.1% of deaths below 70 and 46.9% of total deaths above 70 years due to CVDs, making it a severe public health issue [62].

We found few studies that looked at the temperature-mortality association in India; however, none were from Puducherry or the surrounding states. The findings from these studies are instrumental in highlighting the differences in regional temperature distribution ranges within India and the corresponding MMT for all-cause mortality, which ranged from 28.6 °C (temperature range 15.3°- 33.2 °C) to 30 °C (pan India temperature range 0.4 °C—~ 40 °C) [22, 63]. Further studies are needed to characterize the micro-climatic, demographic and socio-cultural differences in temperature attributable, cause-specific mortality.

While there are relatively fewer “heat wave” days in Puducherry, if the warming trend continues as projected, the temperatures for Puducherry could increase or lead to erratic extreme temperatures. It could lead to either a potential right-shift of the optimal temperatures, if this occurs gradually, or a significant increase in the AF for CVD mortality due to hot temperatures if there are more erratic extreme days. For example, a study from Hyderabad, a city with higher mean temperatures than Puducherry, found an increase in all-cause mortality by 16% and 17% for maximum temperatures above 40 °C and heat index > 54 °C respectively [64]. The pattern of anthropogenic climate change over India is a complex one. Mean temperatures in the South Asian region have been decreasing in the past decades and India has not seen an increase in the maximum temperature trends since the 1970s [36, 65]. From a health perspective, T_app_, which accounts for humidity, is better at measuring the health effects. The increase in humidity in India has led to T_apps_ increasing in India and thereby the severity and occurrence of heat has increased [65]. In the future, pollution control measures and a slower pace of irrigation expansion will likely counter the present cooling effects being seen and as humidity is projected to increase, the net effect will be a gradual rise in hot temperatures, especially during heat waves [65]. It is difficult to assess whether the rise in temperatures might be accompanied by a decrease in the AF for cold-related mortalities or whether only the severity and frequency of heat waves will increase. The absolute number of CVD mortalities attributable to non-optimal temperatures are likely to increase, however, since more people will be at risk or have CVDs in the future.

A recent multi-country, multi-community study found that most excess deaths occur in eastern/southern Asia, especially in coastal cities, highlighting the difficulties to protect, react and reduce adverse temperature effects in these regions, partially due to the large and dense population [36]. As there are several large cities both within this region and along the extensive coastline of India, it is imperative that further research is done on how temperature affects the health of the local population. There is also a need to develop a tailored temperature-health impact management and adaptation plan to reduce the burden of CVD mortalities due to non-optimal temperatures that accounts for regional demographics. These preliminary estimates can be used as a basis to support further detailed research on this topic in Puducherry or elsewhere.

### Strengths

Our study has several strengths. First, it demonstrates how both relatively cold and hot temperatures affect CVD mortality in the tropical region of Puducherry. The high quality of patient level data allowed for examining the effects of age-and-sex grouped together, which has not been explored in the Indian context. It highlights the added vulnerability of older women to extreme heat. Second, the case-crossover approach adjusted for stable within subject and residual individual confounders, particularly from variables that may not have been recorded, by design and allowed us to preserve individual characteristics. We could thus conduct individual-level and inter-individual analysis through subgrouping. Third, this is the first study of its kind in this region; we were able to show how regional and demographic variations play an important role in determining the fraction of CVD mortalities attributable to non-optimal temperatures over a relatively long time period. Additionally, the small size of Puducherry coupled with a single multi-speciality state government hospital and robust health system means that we were able to capture the general trends from the main state government hospital, which caters to majority of the population within Puducherry. Finally, we were able to demonstrate that cold temperatures have a large AF consistent with other Indian studies as shown. Overall, our study is comparable to global studies from different climate zones and areas, implying a greater contribution of population, genetics and acclimatization to the temperature-CVD mortality relationship. The results from our sensitivity analyses using only 5-year data from the whole hospital, or changing the knot placement were all insensitive to the changes in the model, supporting the robustness of our findings about the association in Puducherry.

### Limitations

The study has several limitations that we offer for consideration. The small sample size which we managed to obtain and the variability in daily in-hospital mortality reduces the certainty with which we can draw inferences, particularly regarding subgroups. This is also shown by the wide CIs, especially at the extreme ends. Since the data stems from a state-run government hospital, we cannot account for patients who might have chosen to seek treatment in a private hospital or travelled to neighbouring states, however as mentioned above this is likely to be minimal. We also did not include air pollution in our study due to lack of suitable data. Thus, we cannot evaluate whether air pollutants are correlated to temperature in our study area, as seen in some studies. This implies that our association with temperature may also include air pollution effects to the extent temperature affects air pollution levels. However, it must be noted that temperature has been shown to have a relationship that is independent from the effects of air pollution [66, 67]. Owing to its geographical location, with ventilating effects from the sea and land breeze, and relatively low population density, Puducherry levels of air pollutants are relatively low with an average mean of about 30–49 µg/m^3^ for PM10 and a declining trend since 2016 [68].This study assumes that the effects of temperature on CVD mortality are through an acute exposure (the effect on CVD is assumed to only happen over the 21 day-lag that was modelled) as opposed to the chronic nature of temperature exposure. One of the key assumptions of this study was that temperature changes recorded at weather stations affected the entire population in the same way. Different parts of the district may have been more insulated from these changes. As we did not have information on the specific location of individuals prior to admission it was not possible to explore this in more detail, but we felt as though differences were unlikely to be sufficiently systematically different to confound the association between temperature and CVD admissions as shown in Figure S11. Finally, as there is no way to separate the temperature effects on the CV system from the medical interventions or hospital conditions that might counter the actual effects and work to prolong life, used the outcome of ‘fatal admission’ as opposed to simply mortality, as is commonly used. This allowed us to assume that exposure lasted only until hospital admission, following which treatments and climate control in the hospital could be expected to modify the exposure-mortality association, especially since patients who were admitted for more than 48 h spent an average of 7 days in hospital before dying. We have performed sensitivity analysis including only those who spent a maximum of 10 days in hospital (Supplement Fig. 7). While we tried to assess the cause-specific risk of morality, there were some limitations, for example as most of the patients presented with multiple CVDs, the overall risk from individual CVDs cannot be confidently assessed and there is likely a mixed effect.

## Conclusion

Our study highlighted the burden of CVD mortality within hospitals attributable to cold and hot non-optimal temperatures in Puducherry. We found the MMT for Puducherry differs from the MMT reported at the national level, pointing towards a need to follow up with larger, regional studies. There are also age-related sex differences in the vulnerability of the population to non-optimal temperatures If the warming trend over India continues, heat will likely become a bigger challenge for public health, particularly increasing the vulnerability of females. As such, public health interventions need to be contextually and gender tailored for the local population and need to address the cold-impacts as well as the heat-impacts, even in tropical regions. In addition, our findings suggest the importance of considering optimal temperatures as the measure against which ‘cold’ and ‘hot’ are refined for a region.

Finally, we feel that there needs to be an increased awareness about the health impacts of climate change. Research is one of the main ways to raise awareness and ensure measures such as healthcare system preparedness, early warning systems, climate resilient infrastructural developments and urban planning are taken. It can also contribute to the development or enhancement of climate informed health policies. There is also an urgent need to have central, individual level, health registers which can be accessed for research. Overall, our findings contribute to understanding how climatic conditions can affect CVD outcomes in India.

## Supplementary Information

**Additional file 1: Table S1.** CVD classification code system used in this study. ICD-10 codes (given in brackets) have been adapted to form the categories used in this study. We divided the CVDs into 3 broad categories, namely ischemic heart diseases, cerebrovascular accidents, and other heart diseases, which included 7 sub-categories, **Table S2.** Distribution of climate variables based on population characteristics, **Figure S1.** Comparison of the T_app_-mortality association in models with varying knot placements. Model 1 has 2 equally placed knots, model2 has 3 knots at the 5^th^, 50^th^ and 95^th^ percentile of the T_app_ model3 has 3 knots at the 25^th^, 50^th^ and 75^th^ percentile and model 4 has 2 knots on the 5^th^ and 95^th^ percentile of the T_app_, **Figure S2.** Individual exposure-response associations for the 4 models depicted in figure S1, **Figure S3.** Comparison of the exposure-response association assuming either a Quasi-poisson or conditional logistic regression with binomial likelihood, such as the one we used, **Figure S4.** Comparison of the exposure-response association using the complete 10 year data set with cases only from the cardiology department for 2011-2015 and from both the cardiology department and all other departments for 2016-2020 vs using only 5 year data with cases from all the departments from 2016-2020, **Figure S5.** Annual trends in CVD admissions and mortality, **Figure S6.** 3D- model depicting the RR for the lagged exposure-response association, **Figure S7.** Comparison of the exposure-response association using the complete 10 year data set vs restricting it to patients who spent less than 10 days in hospital. The black line depicts the overall association while the grey line depicts the patients who spent less than 10 days admitted to hospital before dying, **Figure S8.** Exposure-response association at different lag days. a.) 1 day, b.) 5 days, c.) 10 days and d.) 20 days, **Figure S9.** The role of co-morbidities in the exposure-response association. a.) population with co-morbidities and b.) Population without co-morbidities, **Figure S11.** Comparison of daily T_app_ values from the two weather stations we used in our analysis, namely Cuddalore and Puducherry, **Figure S12.** Comparison of daily T_app_ values from the two weather stations we used in our analysis, namely Cuddalore and Puducherry, along with the average of both which we used in our model (depicted in black).

## Data Availability

The meteorological data used in this study is available from the Indian Meteorological Department, upon request, from their Climate Data Service Portal (https://cdsp.imdpune.gov.in/). The health data that support the findings of this study are available from the Puducherry Department of Health following their prescribed procedures and ethical approval as this data is not publicly available. A sample of the dataset can be available from the authors upon reasonable request and with permission of the Puducherry Department of Health with ethical permissions from an Indian ethics review committee. R codes are available from the corresponding author upon request.

## Abbreviations

AF: Attributable fraction
CV: Cardiovascular
CVA: Cerebrovascular accidents (also commonly referred to as cerebrovascular disease)
CVD: Cardiovascular diseases
Dlnm: Distributed lag non-linear model
hPa: Vapour pressure
ICD-10: International classification of diseases
IGGGH: Indira Gandhi Government General Hospital
IHD: Ischemic heart disease
IMD: Indian Meteorological Institute
LMIC: Low and middle income countries
MMT: Minimum mortality temperature
NCDs: Non-communicable diseases
RCP: Regional concentration pathway
RH: Relative humidity
Ta: Dry bulb temperature
T_app_: Apparent temperature
Tmax: Maximum temperature
Tmin: Minimum temperature
UT: Union Territory
WS: Wind speed
