# Correction: Non-optimal apparent temperature and cardiovascular mortality: the association in Puducherry, India between 2011 and 2020

**DOI:** 10.1186/s12889-024-20143-2

**Published:** 2024-09-27

**Authors:** Shreya S. Shrikhande, Hugo Pedder, Martin Röösli, Mohamed Aqiel Dalvie, Ravivarman Lakshmanasamy, Antonio Gasparrini, Jürg Utzinger, Guéladio Cissé

**Affiliations:** 1 Swiss Tropical and Public Health Institute, Kreuzstrasse 2, Allschwil, CH-4123 Switzerland; 2 University of Basel, Basel, Switzerland; 3 Population Health Sciences, University of Bristol, Bristol, UK; 4 School of Public Health and Family Medicine, Centre for Environmental and Occupational Health Research, University of Cape Town, Cape Town, South Africa; 5 State Surveillance Officer, Department of Health and Family Welfare Services, Govt. of Puducherry, Puducherry, India; 6 Department of Public Health, Environments and Society, London School of Hygiene and Tropical Medicine, London, UK; 7 Centre On Climate Change and Planetary Health, London School of Hygiene and Tropical Medicine, London, UK; 8 Centre for Statistical Methodology, London School of Hygiene and Tropical Medicine, London, UK

**BMC Public Health (2023) 23:291**

10.1186/s12889-023-15128-6

The original publication of this article contained an incorrect author name. The incorrect and correct information is listed in this correction article, the original article has been updated.

Incorrect

Mohammad Aqiel Dalvie

Correct

Mohamed Aqiel Dalvie

