# Supplementary material for: Non-optimal apparent temperature and cardiovascular mortality: the association in Puducherry, India between 2011 and 2020
Source: BMC Public Health. 2023 Feb 8;23:291. doi: 10.1186/s12889-023-15128-6 (PMC9909923; doi:10.1186/s12889-023-15128-6)
Supplement: Supplementary file 1 — Additional file 1: Table S1. CVD classification code system used in this study. ICD-10 codes (given in brackets) have been adapted to form the categories used in this study. We divided the CVDs into 3 broad categories, namely ischemic heart diseases, cerebrovascular accidents, and other heart diseases, which included 7 sub-categories, Table S2. Distribution of climate variables based on population characteristics, Figure S1. Comparison of the Tapp-mortality association in models with varying knot placements. Model 1 has 2 equally placed knots, model2 has 3 knots at the 5th, 50th and 95th percentile of the Tapp model3 has 3 knots at the 25th, 50th and 75th percentile and model 4 has 2 knots on the 5th and 95th percentile of the Tapp, Figure S2. Individual exposure-response associations for the 4 models depicted in figure S1, Figure S3. Comparison of the exposure-response association assuming either a Quasi-poisson or conditional logistic regression with binomial likelihood, such as the one we used, Figure S4. Comparison of the exposure-response association using the complete 10 year data set with cases only from the cardiology department for 2011-2015 and from both the cardiology department and all other departments for 2016-2020 vs using only 5 year data with cases from all the departments from 2016-2020, Figure S5. Annual trends in CVD admissions and mortality, Figure S6. 3D- model depicting the RR for the lagged exposure-response association, Figure S7. Comparison of the exposure-response association using the complete 10 year data set vs restricting it to patients who spent less than 10 days in hospital. The black line depicts the overall association while the grey line depicts the patients who spent less than 10 days admitted to hospital before dying, Figure S8. Exposure-response association at different lag days. a.) 1 day, b.) 5 days, c.) 10 days and d.) 20 days, Figure S9. The role of co-morbidities in the exposure-response association. a.) population with co [file 12889_2023_15128_MOESM1_ESM.docx]

# Supplement

Table S1: CVD classification code system used in this study. ICD-10 codes (given in brackets) have been adapted to form the categories used in this study. We divided the CVDs into 3 broad categories, namely ischemic heart diseases, cerebrovascular accidents, and other heart diseases, which included 7 sub-categories.

|  | **Mean** | **Minimum** | **1^st^ Quartile** | **Median** | **3^rd^ Quartile** | **Maximum** |
| --- | --- | --- | --- | --- | --- | --- |
| **Apparent temperature (°C)** | | | | | | |
| **Male** | 33.25 | 23.25 | 30.66 | 33.82 | 35.95 | 40.60 |
| **Female** | 33.43 | 23.25 | 30.92 | 34.01 | 36.07 | 40.60 |
| **Less than 48** | 33.32 | 24.01 | 30.83 | 33.86 | 36.0 | 40.60 |
| **More than 48** | 33.32 | 23.25 | 30.77 | 33.90 | 36.09 | 40.60 |
| **Comorbidities** | 33.42 | 24.30 | 30.98 | 34.0 | 36.07 | 40.60 |
| **Cerebrovascular accidents** | 33.37 | 23.25 | 30.85 | 33.95 | 36.14 | 40.60 |
| **Other CVD types** | 33.20 | 24.41 | 30.60 | 33.81 | 35.94 | 40.60 |
| **Ischemic heart disease** | 33.34 | 23.25 | 30.86 | 33.90 | 36.10 | 40.60 |
| **Average Temperature (°C)** | | | | | | |
| **Male** | 28.40 | 21.25 | 26.30 | 28.45 | 30.45 | 35.15 |
| **Female** | 28.55 | 21.25 | 26.45 | 28.70 | 30.65 | 36.0 |
| **Less than 48** | 28.47 | 23.05 | 26.35 | 28.55 | 30.55 | 35.15 |
| **More than 48** | 28.47 | 21.25 | 26.30 | 28.60 | 30.60 | 35.10 |
| **Comorbidities** | 28.46 | 21.25 | 26.35 | 28.55 | 30.55 | 36.0 |
| **Cerebrovascular accidents** | 28.49 | 21.25 | 26.35 | 28.65 | 30.60 | 35.10 |
| **Other CVD types** | 77.04 | 22.70 | 26.25 | 28.45 | 30.45 | 36.0 |
| **Ischemic heart disease** | 28.52 | 21.25 | 26.35 | 28.55 | 30.65 | 36 |
| **Humidity (%)** | | | | | | |
| **Male** | 77.06 | 43.0 | 72.75 | 77.25 | 81.75 | 100.0 |
| **Female** | 76.68 | 47.25 | 73.0 | 77.0 | 81.50 | 100.0 |
| **Less than 48** | 76.80 | 43.0 | 72.50 | 77.0 | 81.50 | 100.0 |
| **More than 48** | 76.79 | 47.25 | 72.50 | 77.0 | 81.50 | 100.0 |
| **Comorbidities** | 76.89 | 43.0 | 72.75 | 77.0 | 81.50 | 100.0 |
| **Cerebrovascular accidents** | 76.69 | 47.75 | 72.50 | 77.0 | 81.50 | 99.0 |
| **Other CVD types** | 77.04 | 43.0 | 73.0 | 77.12 | 81.50 | 100.0 |
| **Ischemic heart disease** | 76.70 | 43.0 | 72.50 | 77.0 | 81.50 | 100.0 |

Table S2: Distribution of climate variables based on population characteristics

Sensitivity analysis


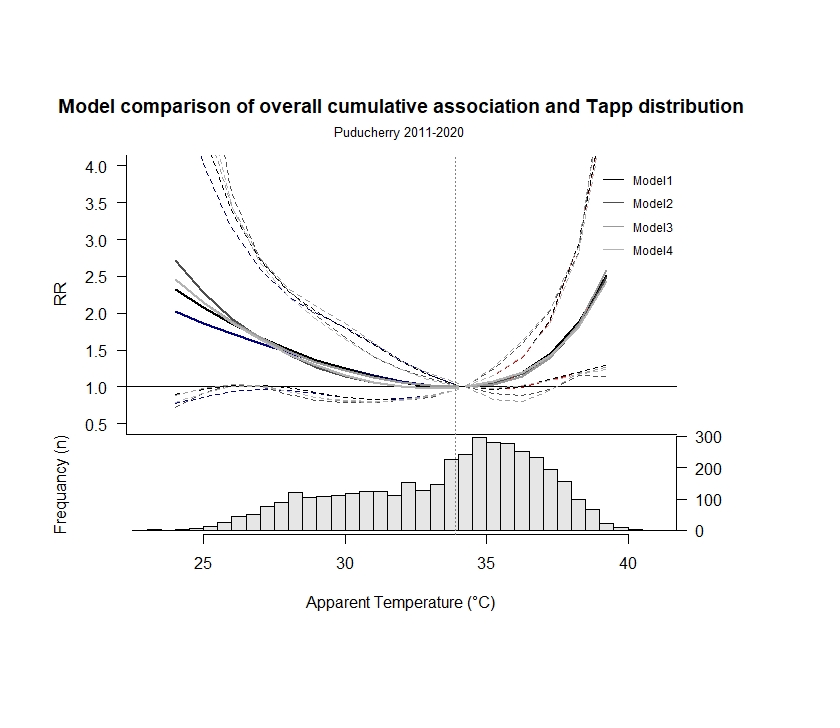
All models are compared to the model used in the final analysis. We changed the placement of the exposure-response knots as shown in Figure S1 and S2. Here, model 1 has 2 equally placed knots, model 2 has 3 knots at the 5^th^, 50^th^ and 95^th^ percentile of the T_app_ model3 has 3 knots at the 25^th^, 50^th^ and 75^th^ percentile and model 4 has 2 knots on the 5^th^ and 95^th^ percentile. There is no significant difference between models.

Figure S1: Comparison of the T_app_-mortality association in models with varying knot placements. Model 1 has 2 equally placed knots, model2 has 3 knots at the 5^th^, 50^th^ and 95^th^ percentile of the T_app_ model3 has 3 knots at the 25^th^, 50^th^ and 75^th^ percentile and model 4 has 2 knots on the 5^th^ and 95^th^ percentile of the T_app._


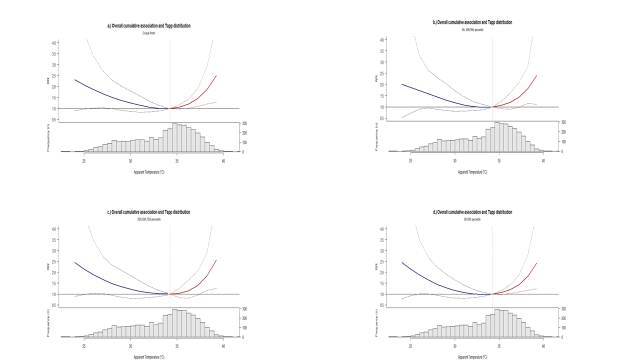


Figure S2: Individual exposure-response associations for the 4 models depicted in figure S1.


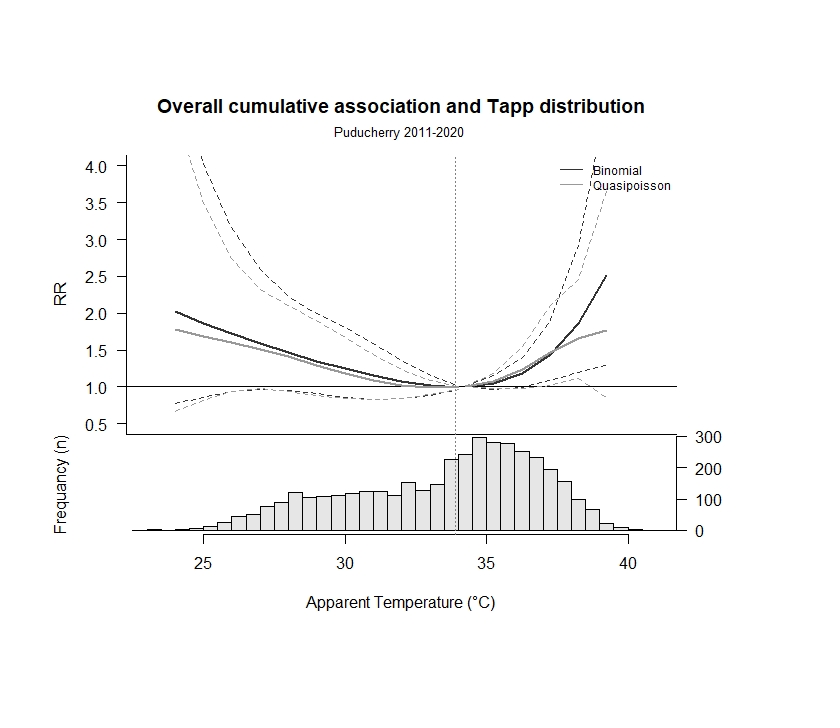
We repeated the analysis comparing our final model fitted with a negative binomial likelihood to one with a quasi-Poisson likelihood as shown in Figure S3, as is commonly used in many studies. The two models typically produce very similar results, but in the quasi-Poisson the variance is a linear function of the mean, whilst in the negative binomial model it is a quadratic function of the mean, allowing for slightly more flexibility in the specification of the variance. We found the associations to be similar, suggesting that our findings were not sensitive to the choice of likelihood.

Figure S3: Comparison of the exposure-response association assuming either a Quasi-poisson or conditional logistic regression with binomial likelihood, such as the one we used


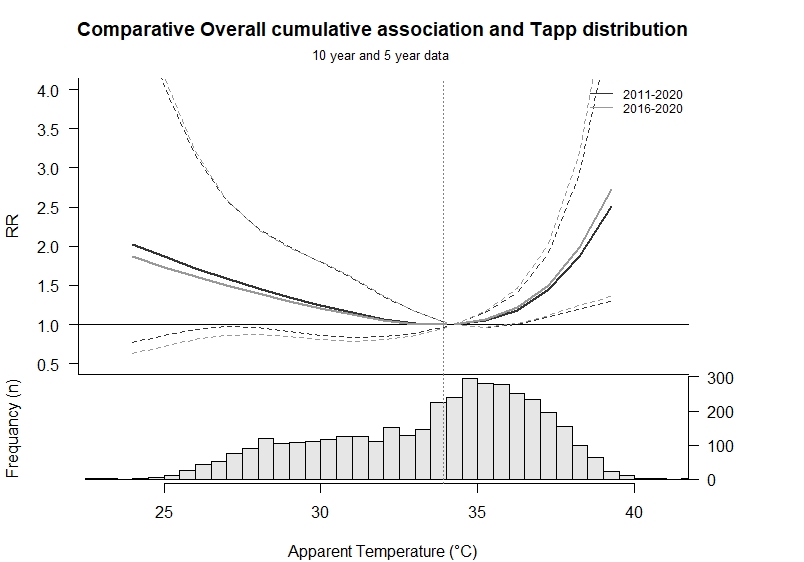
We repeated the analysis using data from only 2016-2020, which had data across the entire hospital. As seen in Figure S4, there does not appear to be a significant difference between the associations.

Figure S4: Comparison of the exposure-response association using the complete 10 year data set with cases only from the cardiology department for 2011-2015 and from both the cardiology department and all other departments for 2016-2020 vs using only 5 year data with cases from all the departments from 2016-2020.

Figure S5 shows the annual trends in the monthly hospital admission and mortality from CVDs. A.) shows the annul CVD mortality while b.) shows the CVD mortality relative to the total mortality. C.) shows the annual CVD admissions while d.) shows the annual CVD admissions relative to the total admissions. As can be seen, there is an increase in both the hospital admissions and mortalities from CVDs over the past 10 years.


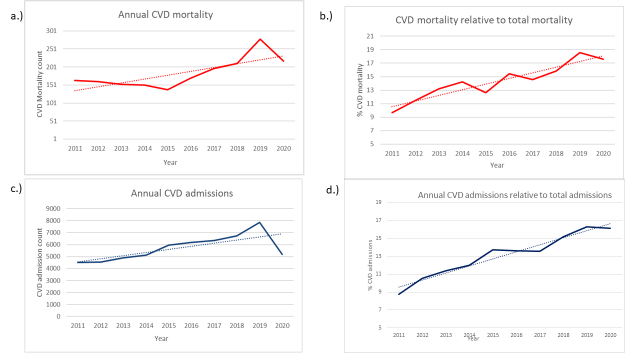


Figure S5: Annual trends in CVD admissions and mortality


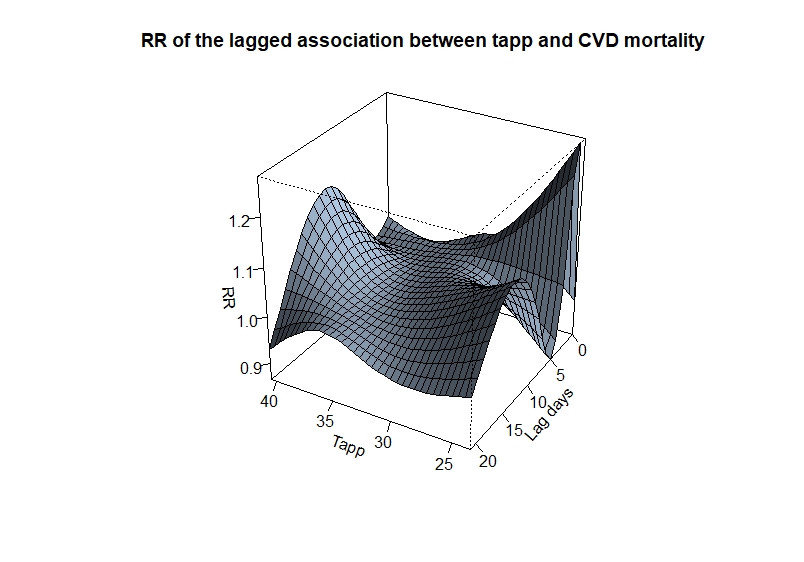
Figure S6 shows the 3-D plot of the lagged association between T_app_ and CVD mortality in Puducherry. The temperature-CVD mortality association shows a non-linear pattern. The 3-D view of this relationship shows that the risks of in-hospital CVD mortality attributed to temperature has a temporal distribution.

Figure S6: 3D- model depicting the RR for the lagged exposure-response association.

We see that cold temperature has an almost immediate response or increase in RR while hot temperatures show a delayed association by about 5 days. The cold effect peaks at day 1 before gradually decreasing below 1 around lag day 5. The cold-CVD mortality association risk increases slightly from around day 9 to day 16 where it peaks at day 11. Hot temperatures show a delayed response with the risk of CVD-mortality only seen after 5 lag days which persists for 16 days. This risk is relatively lesser compared to the cold-CVD mortality risk for 5 lag days.


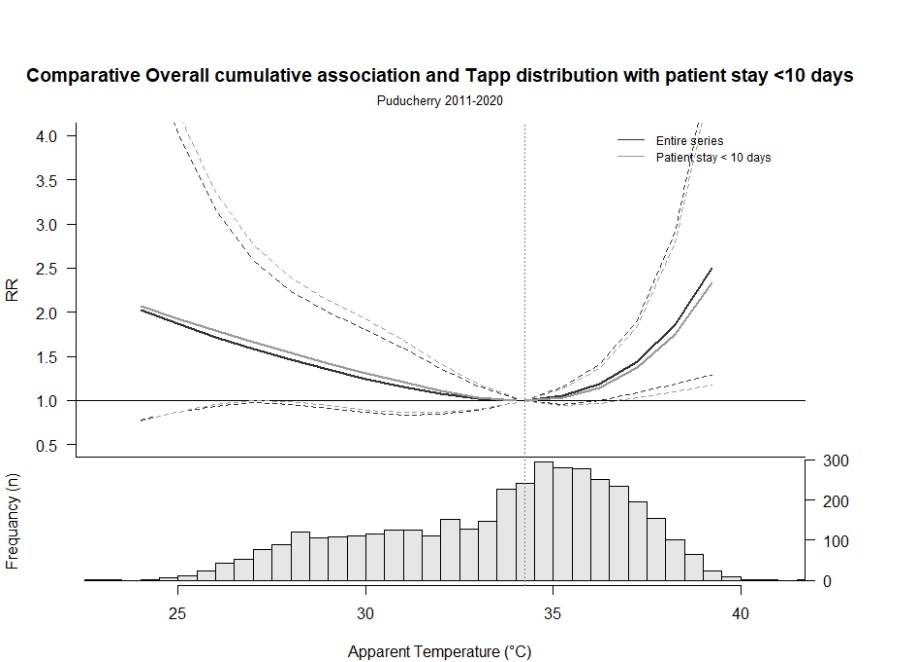
Figure S7 shows the results of the sensitivity analysis comparing all the patients versus patients who were admitted for less than 10 days before dying. The results show that there is relatively no difference in the association between patients who spent less than 10 days and the association for all patients.

Figure S7: Comparison of the exposure-response association using the complete 10 year data set vs restricting it to patients who spent less than 10 days in hospital. The black line depicts the overall association while the grey line depicts the patients who spent less than 10 days admitted to hospital before dying.

Figure S8 shows the RR of CVDs at lag day 1, 5, 15 and 20. Cold has an immediate outcome at lag day 1 which attenuates around day 5 as heat starts to have an effect. The effects of both hot and cold temperatures last for a bout 15 days.


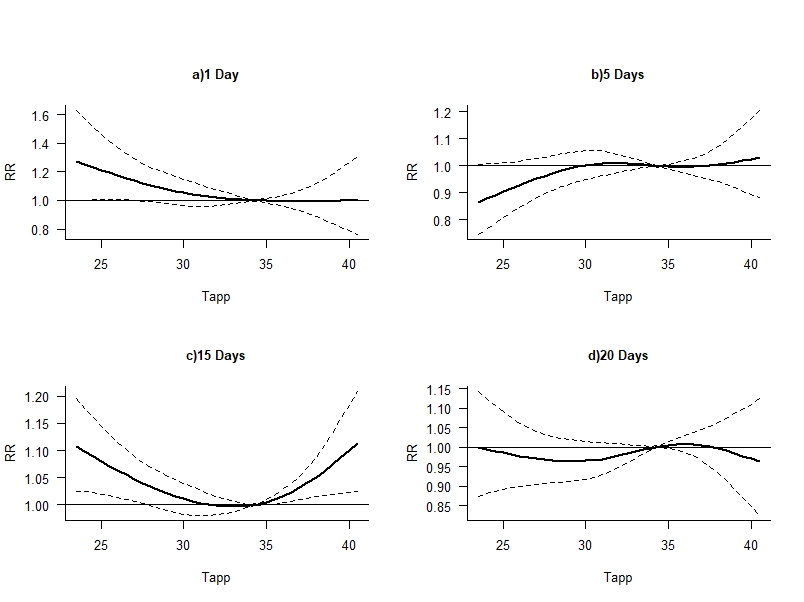


Figure S8: Exposure-response association at different lag days. a.) 1 day, b.) 5 days, c.) 10 days and d.) 20 days.


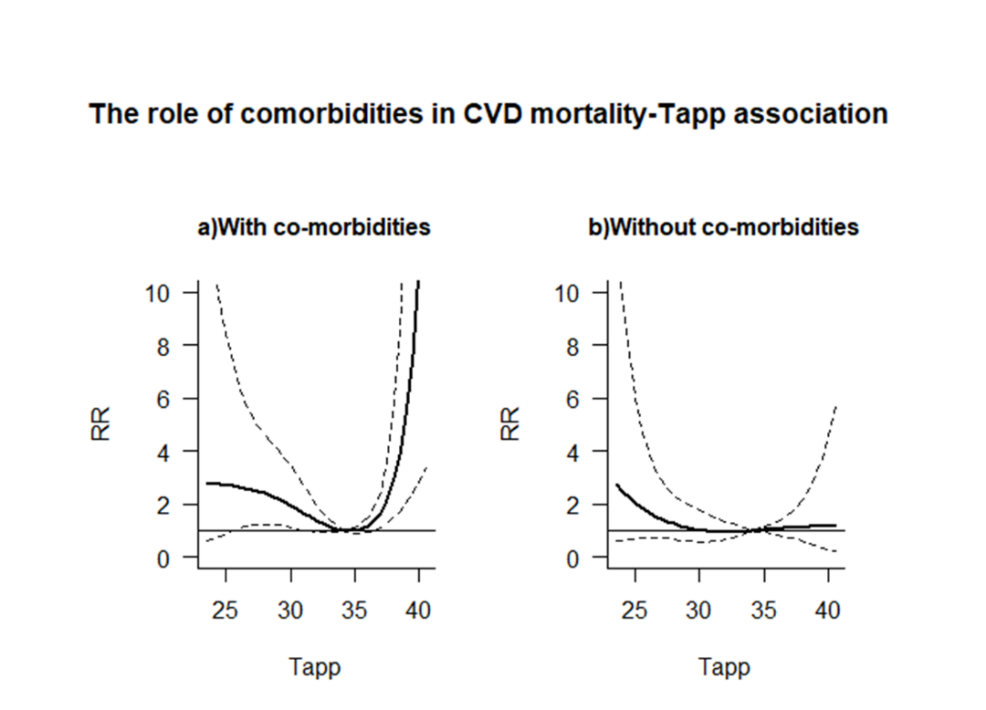
Figure S9 shows the RR for temperature associated CVD mortalities between people with and without co-morbidities such as hypertension, diabetes and alcoholism. People with co-morbidities appear to be more vulnerable to the effects of non-optimal temperatures than those without.

Figure S9: The role of co-morbidities in the exposure-response association. a.) population with co-morbidities and b.) Population without co-morbidities.


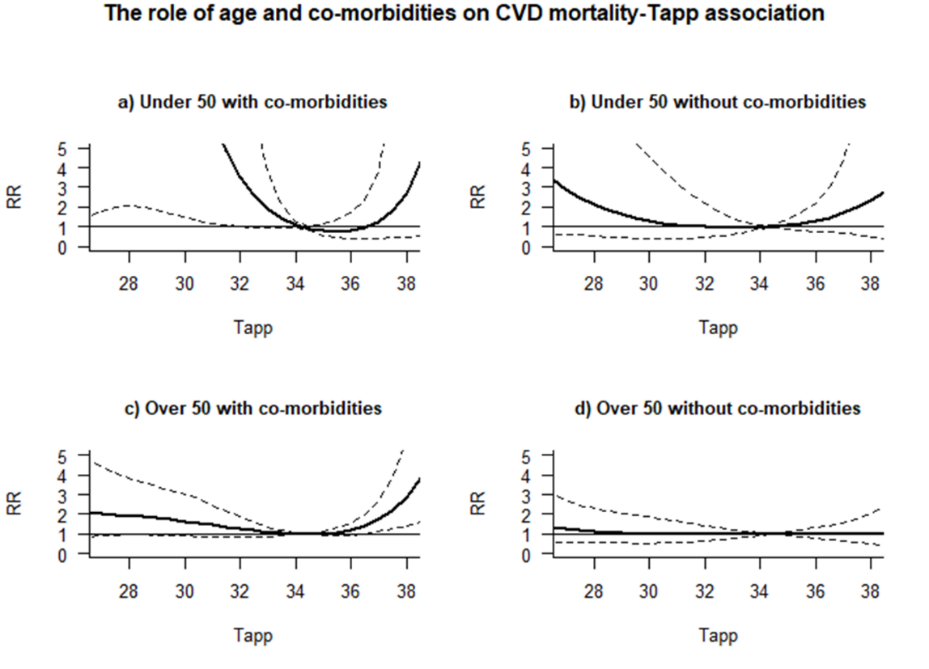
This association was further stratified by age groups as shown in Figure S10. All groups were vulnerable to non-optimal temperatures except for those over 50 without co-morbidities. We recommend further studies to better understand this phenomenon.

Figure S10: The role of age and co-morbidities in the exposure-CVD mortality association. a.) Population under 50 with co-morbidities, b.) Population under 5o without co-morbidities, c.) Population over 5 with co-morbidities and d.) Population over 50 without co- morbidities. Graph restricted to the central 95t percentile of the T_app_ distribution due to wide Cis at extreme ends.

Figures S11 shows a comparison of the daily T_app_ values from individual stations. We used an average value of data from both substations in our analysis, which has been presented in Figure S12.


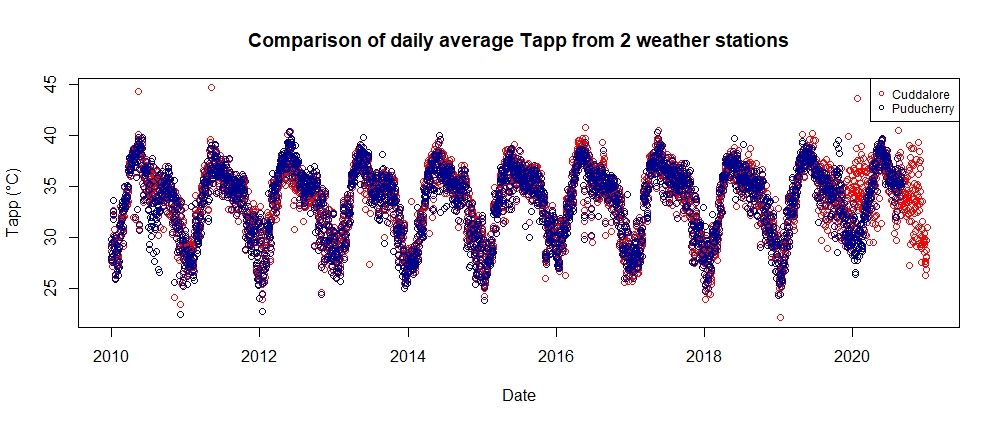


Figure S11: Comparison of daily T_app_ values from the two weather stations we used in our analysis, namely Cuddalore and Puducherry.


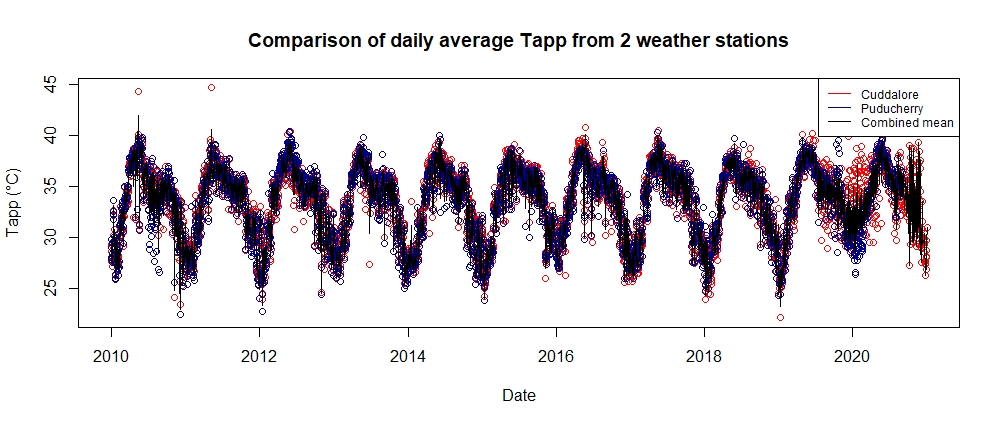


Figure S12: Comparison of daily T_app_ values from the two weather stations we used in our analysis, namely Cuddalore and Puducherry, along with the average of both which we used in our model (depicted in black).
